# Beyond in-phase and anti-phase coordination in a model of joint action

**DOI:** 10.1007/s00422-016-0691-9

**Published:** 2016-06-08

**Authors:** Daniele Avitabile, Piotr Słowiński, Benoit Bardy, Krasimira Tsaneva-Atanasova

**Affiliations:** Centre for Mathematical Medicine and Biology, School of Mathematical Sciences, University of Nottingham, University Park, Nottingham, NG7 2RD UK; Department of Mathematics, College of Engineering, Mathematics and Physical Sciences, University of Exeter, Exeter, Devon EX4 4QF UK; EuroMov, Montpellier University, 700 Avenue du Pic Saint-Loup, 34090 Montpellier, France; Institut Universitaire de France, Paris, France; EPSRC Centre for Predictive Modelling in Healthcare, University of Exeter, Exeter, Devon EX4 4QF UK

**Keywords:** Coupled oscillators, Dynamical system, Bifurcation analysis, Coordination regimes, Numerical continuation, Parameter dependence

## Abstract

In 1985, Haken, Kelso and Bunz proposed a system of coupled nonlinear oscillators as a model of rhythmic movement patterns in human bimanual coordination. Since then, the Haken–Kelso–Bunz (HKB) model has become a modelling paradigm applied extensively in all areas of movement science, including interpersonal motor coordination. However, all previous studies have followed a line of analysis based on slowly varying amplitudes and rotating wave approximations. These approximations lead to a reduced system, consisting of a single differential equation representing the evolution of the relative phase of the two coupled oscillators: the HKB model of the relative phase. Here we take a different approach and systematically investigate the behaviour of the HKB model in the full four-dimensional state space and for general coupling strengths. We perform detailed numerical bifurcation analyses and reveal that the HKB model supports previously unreported dynamical regimes as well as bistability between a variety of coordination patterns. Furthermore, we identify the stability boundaries of distinct coordination regimes in the model and discuss the applicability of our findings to interpersonal coordination and other joint action tasks.

## Introduction

Many body movements are periodic in their nature [18]. For example, postural sway [2], walking [8, 20], running [37], swimming [54], galloping [8, 20] and juggling [58] have a cyclic pattern in the position of the end effectors or joint angles. Synchronisation is a fundamental aspect of oscillatory coordination dynamics in human and animal body movements [33] and has been found in many different situations [48]. Coordination is characterised by a bounded temporal relationship created by a convergent dynamical process [26, 44]. Coordination regimes depend on symmetries and couplings between oscillators. Frequency entrainment, where two oscillators adopt a central frequency, occurs even with a very weak coupling. With a relatively strong coupling or if the system is symmetrical, phase entrainment can also take place. These processes may be continuous or intermittent; that is, the phases of the two oscillators may also align periodically [18, 35, 47].

In the case of two coupled oscillators, the regular patterns of coordination are well captured by the properties of the relative phase between the periodic movements of the two subsystems [32, 33]. The simplest pattern is observed when the phase of the two oscillators coincide to give in-phase ($0^{\circ }$) monostable coordination pattern. An example of this behaviour is given by iso-lateral limb movements [6]. Monostable anti-phase ($180^{\circ }$) coordination can also occur, and an example of such behaviour is observed in team sports (competitive games) [5, 10, 12, 15]. In many real systems, anti-phase stability coexists with in-phase stability [22, 33, 44, 56]. Previous studies address the modelling of the two coupled oscillators as a nonlinear dynamical system, the fitting of its periodic orbits to human movements [28] and the systematic analysis of the effects of linear and nonlinear terms to the observed limit cycles [4]. The observed relations between frequency and amplitude [22] as well as peak velocities [44] in many but not all [3] oscillatory movements turned out to be well represented by a hybrid oscillator [22] formed by a combination of Van der Pol and Rayleigh nonlinear damping terms.

A classical example of model exhibiting bistability is the so-called HKB model proposed in the seminal work by Haken, Kelso and Bunz [18, 22]. The model, which was originally developed for bimanual finger coordination [30], was found to be representative of a wide range of applications in human movement [7, 18], suggesting that the dynamics that it produces are somehow fundamental and make formal construct for the study of coordination dynamics [32, 33]. Although the model was originally developed in order to account for intra-personal phenomena, the same patterns have been shown to be representative of both sensorimotor and interpersonal behaviours [31, 49, 50]. The model successfully reproduces not only the patterns of stability observed in bimanual coordination experiments but also their dependence upon frequency [22]. The HKB model admits a potential function that yields the experimentally observed change in attractors’ landscapes. Furthermore, the HKB model and its stochastic extension reproduced the characteristic fluctuation increase and slowing down observed experimentally near instabilities [51].

The development of the HKB model has been inspired by the in-phase and anti-phase coordination dynamics observed in bimanual coordination in the context of the finger movements experiment [30, 52, 53]. Therefore, most previous research has focused on a fixed set of model parameters that guarantees the stability of these particular dynamics. Furthermore, significant contributions to understanding these coordination patterns (albeit in a narrow parameter range and with limiting assumptions on the parameters controlling the coupling strength) have been made for different oscillator frequencies and inputs [1, 3, 7, 17, 19, 25] as well as noise in the system [9, 49, 50]. All previous mathematical analyses of the HKB model have focused on the relative phase dynamics, under the assumption that the amplitude of the coupled oscillators is constant [1, 3, 9, 17, 19, 22]. Several recent articles have studied the phase-approximation dynamics in the HKB model by considering the multiple stable states of the system and the ability to switch between them by changing the frequency and the coupling parameters [38]. The bifurcations leading to transitions between anti-phase and in-phase dynamics in a reduced phase approximation of the HKB model [16] have been also studied. To our knowledge, however, a bifurcation analysis of the full four-dimensional HKB model, considering all model parameters as well as general (i.e. weak and strong) coupling strengths, has not been performed. Such analysis could provide an insight into other possible qualitative behaviours that the solutions of the model might exhibit, as well as characterise the possible changes in the dynamics of the solutions corresponding to any changes in the parameter values of the model. We also note recent further developments of dynamical systems’ approaches for studying sensorimotor dynamics, involving dynamical repertoires, hierarchies of timescales and structured flows on manifolds [23].

Given that the HKB model is a widely accepted tool in this field, it is imperative to examine systematically all the possible coordination regimes supported by this system. In addition, classifying changes of dynamical regimes in terms of positions and velocities of the two coupled oscillators would undoubtedly shed light on the HKB model’s applicability to explain movement coordination in joint actions and human interactions with an adaptive virtual partner (VP)[13, 34, 40, 59–61]. In the present paper, we take a different approach in analysing the HKB model, as we study the full four-dimensional system of first order differential equations describing the evolution of the positions and velocities of the two coupled oscillators. We begin by characterising the local and global dynamics of the single HKB oscillator and reveal a global transition in the model that governs the existence of periodic solutions in a range of the oscillator’s parameter values. We proceed by systematically characterising the full HKB model dynamics not only by varying the coupling strength parameters but also the rest of the model parameters, i.e. the parameters governing the single oscillator’s properties. In addition to the very well-studied coordination patterns, we find a stable phase-locked solution that spans a wide range of relative phases and persists for a wide range of model parameters’ values. We also show that relaxing the constant amplitude assumption allows for much richer coordination dynamics and coexistence of various stable coordination attractors (multi-stability regimes).

## Results

### Intrinsic properties of the oscillator in the HKB model

Recently, a significant scientific effort has been put towards the development of VP interaction systems. In particular, the single HKB oscillator is being used to drive the movement dynamics exhibited by the VP [13, 34, 59–61]. The dynamics of the model is an important consideration in designing such systems and in particular for parametrising the ordinary differential equation that governs the behaviour of the VP. For example depending on the constraints of the experimental set-up, a certain range of amplitude and/or frequency for the VP periodic behaviour might be desirable. Although some properties of the HKB oscillator have been measured and studied both experimentally and analytically [28, 29], the dynamics of the single HKB oscillator has not been systematically investigated theoretically. To address this gap, we begin by examining a single HKB oscillator:

$$\begin{aligned} \ddot{x} = - \dot{x} \left( \alpha x^2 + \beta \dot{x}^2 - \gamma \right) -\omega ^2 x, \end{aligned}$$

which could be written as a planar autonomous dynamical system of the form:

1 $$\begin{aligned} \dot{x}= & {} y, \nonumber \\ \dot{y}= & {} - y \left( \alpha x^2 + \beta y^{2} -\gamma \right) -\omega ^2 x, \end{aligned}$$

where *x* represents the position, *y* the velocity, $\omega \in \mathbb {R}^+$ is related to the natural frequency of the oscillator and $\alpha , \beta , \gamma \in \mathbb {R}$ are parameters governing the intrinsic dynamics of Eq. (1).

**Fig. 1:**
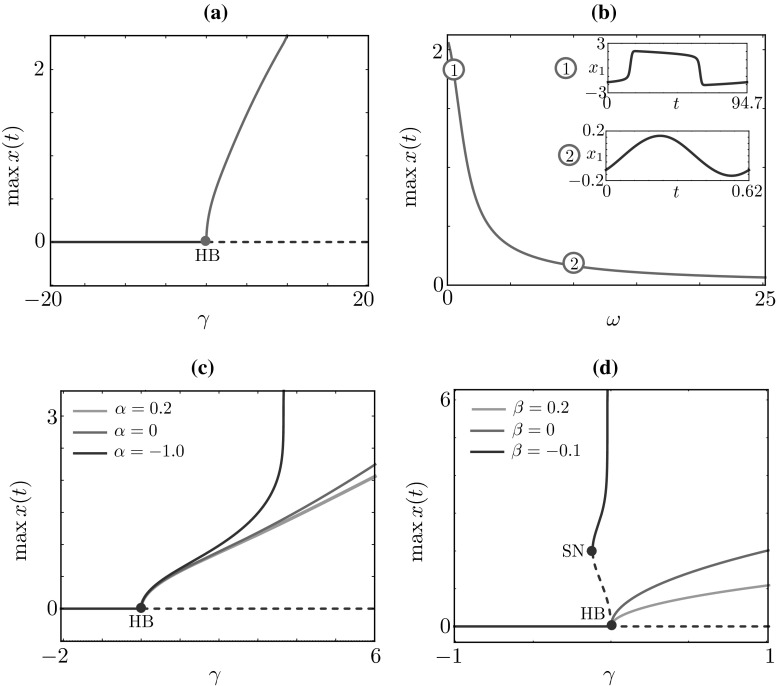
Bifurcation diagrams for a single HKB oscillator. **a** The trivial equilibrium becomes unstable at a supercritical Hopf bifurcation (HB) in the continuation parameter $\gamma$ for $\omega =2$, $\alpha =1$, $\beta =1$. **b** The periodic orbit for $\gamma = 2$ is continued in the parameter $\omega$. The lower $\omega$, the larger the oscillations amplitude and the longer the period. **c**–**d** Continuations in $\gamma$ are repeated for various values of $\alpha$ and $\beta$. In *panel* **c**, for $\alpha =-1$, the periodic branch undergoes a global bifurcation (vertical asymptote), whereas in *panel* **d**, for $\beta = -0.1$, the Hopf bifurcation is subcritical, and the emanating branch restabilises at a saddle-node bifurcation, before disappearing in a global bifurcation. *Solid* (*dashed*) lines represent stable (unstable) states of (1)

The single HKB oscillator is a hybrid Rayleigh–Van der Pol [22] planar system, and although the analysis of planar systems of ordinary differential equations is very well established [21, 27, 43], it has not been applied to the single HKB oscillator model. Furthermore, whenever planar systems are coupled, they are often studied in the weak coupling limit, which we don’t require for the numerical continuation analysis presented here. In our analysis, we focus on the global dynamics of the system and aim to characterise all possible dynamic states that the single HKB oscillator model supports, as well as their dependence on all model parameters. System (1) admits the origin (0, 0) as a trivial steady state for any parameter value $\omega \in \mathbb {R}^+$. Given $\omega >0$, the Jacobian matrix at the trivial equilibrium $(x,y)=(0,0)$ is

$$\begin{aligned} J = \begin{bmatrix} 0&\quad 1 \\ -\omega ^2&\quad \gamma \end{bmatrix} \end{aligned}$$

For $|\gamma | \ge 2\omega$, the Jacobian has a pair of nonzero real eigenvalues:

$$\begin{aligned} \lambda = \frac{\gamma \pm \sqrt{\gamma ^2 - 4\omega ^2}}{2} \end{aligned}$$

Thus, the equilibrium is a stable node (sink) for $\gamma <0$ and unstable node (source) for $\gamma >0$. For $|\gamma | < 2\omega$, the Jacobian has a pair of complex conjugate eigenvalues of the form:

$$\begin{aligned} \lambda = \frac{\gamma }{2} \pm i\frac{\sqrt{4\omega ^2-\gamma ^2}}{2} \end{aligned}$$

Hence, the equilibrium is a stable focus (spiral sink) for $-2\omega<\gamma <0$ and unstable focus (spiral source) for $0<\gamma <2\omega$.

Changing the value of the parameter $\gamma$ near $\gamma =0$ leads to a change in the sign of the eigenvalues’ real part, which is associated with loss or gain of stability. The system undergoes a Hopf bifurcation at $\gamma =0$, which gives rise to oscillations. We could analytically verify further the sufficient conditions for the existence of Hopf bifurcation by showing that:

$$\begin{aligned} \frac{\partial \lambda _{\text {r}}(\gamma )}{\partial \gamma }\Bigg \vert _{\gamma =0}&= \frac{1}{2} \ne 0, \\ l_1(\gamma ) \vert _{\gamma =0}&= \frac{-(\alpha +3\beta \omega ^2 )}{2\omega (\omega ^2+1)} \ne 0 \\&\iff \alpha +3\beta \omega ^2 \ne 0, \end{aligned}$$

where $\lambda _\text {r}$ and $l_1$ are the real part of the eigenvalues and the first Lyapunov coefficient [36], respectively. The sign of the first Lyapunov coefficient [36] determines whether the Hopf bifurcation is subcritical or supercritical; hence, we are in the supercritical (subcritical) case if $\alpha +3 \beta \omega ^2>0$ ($<0$). The system (1) has a degenerate Hopf bifurcation when $\alpha +3 \beta \omega ^2=0$.

Next we carry out bifurcation analysis using numerical continuation in AUTO [11]. We set $\texttt {NTST}=50$, $\texttt {NCOL}=4$ for the mesh, and $\texttt {EPSL}=10^{-9}$, $\texttt {EPSU}=10^{-9}$ for the tolerances of the Newton solver. In Fig. 1a, we continue the trivial steady state $(x,y)=(0,0)$ in $\gamma$: oscillations arise at a supercritical Hopf bifurcation at $\gamma = 0$ since for $\alpha =1$ and $\beta =1$, $\alpha +3 \beta \omega ^2>0$. Stable periodic solutions exist for various values of the intrinsic frequency $\omega$: the lower $\omega$, the larger the oscillations amplitude and the longer the period (see inset in Fig. 1b). Similar scenarios are found for various combinations of $\alpha$ and $\beta$ (Fig. 1c, d). The amplitude of the periodic solutions increase as either $\alpha$ or $\beta$ are decreased. For $\alpha =-1$, the Hopf bifurcation is supercritical and the oscillatory branch is stable, whereas for $\beta =-0.1$ the Hopf bifurcation is subcritical and oscillatory branches, which are originally unstable, restabilise at a saddle node. We note that system (1) exhibits bistability between a stable equilibrium and a stable periodic states in the case of $\beta =-0.1$ (Fig. 1d).

The above analysis reveals that when parameters $\alpha$ and $\beta$ have opposite signs (Fig. 1c, d), there is a critical value for $\gamma$ at which the amplitude (and period) of the stable limit cycle solutions in the model rapidly increase to infinity. As a result, all periodic solutions vanish for values of $\gamma$ above this critical value. Furthermore, such transitions occur robustly for a large range of $\alpha$ and $\beta$ parameter values. We believe it is important to understand where and why this singularity occurs, as it corresponds to a non-physical behaviour. Since this feature has not previously been reported in the literature on the HKB model, we present a thorough investigation of this phenomenon. In order to analyse the behaviour of the system at infinity, we employ methods presented in Chapter 3.10 of reference [46]. We start by projecting system (1) on the Poincaré sphere using the following transformation:

$$\begin{aligned}&X=\dfrac{x}{\sqrt{1+x^2+y^2}}, \;\; Y=\dfrac{y}{\sqrt{1+x^2+y^2}},\\&Z=\dfrac{1}{\sqrt{1+x^2+y^2}}, \end{aligned}$$

which defines one-to-one correspondence between points (*X*, *Y*, *Z*) on the upper hemisphere $\mathrm {S}^2$ with $Z>0$ and points (*x*, *y*) in the plane defined by:

$$\begin{aligned}&x=\dfrac{X}{Z}, \;\; y=\dfrac{Y}{Z}, \end{aligned}$$

**Fig. 2:**
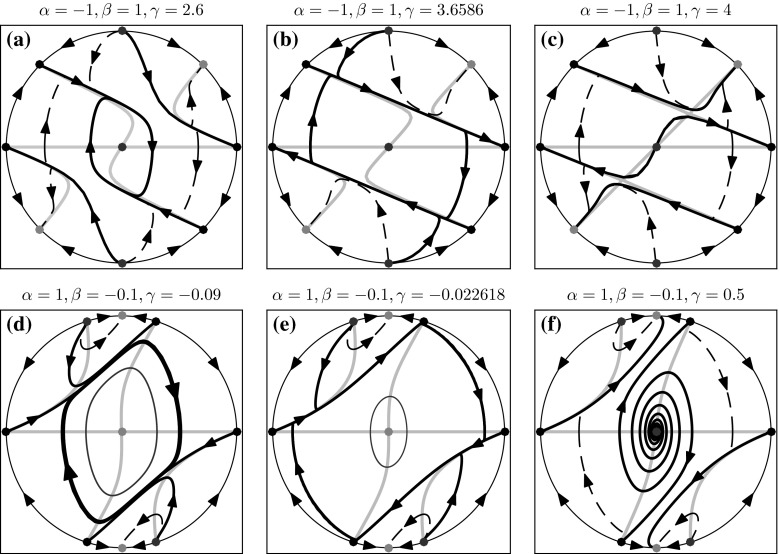
Global phase portraits of the system Eq. (2), projected on the (*X*, *Y*)-plane, for different parameters values. *Green dots* indicate stable equilibria; *red dots* indicate unstable equilibria; *black dots* indicate equilibria of a saddle type; *red line* indicates unstable periodic orbit; *thick black lines* indicate heteroclinic connections between different equilibria or between equilibria and stable periodic orbits; *grey lines* indicate nullclines; *dashed lines* examples of trajectories; *arrows* indicate direction of the flow (colour figure online)

The points on the equator of $\mathrm {S}^2$ correspond to points at infinity of $\mathbb {R}^2$. Under the transformation above, the HKB oscillator on $\mathrm {S}^2$ with $Z>0$ is given by:

2 $$\begin{aligned} \begin{aligned} \dot{X}&=\dfrac{Y}{Z^2} \big [ \alpha X^3 Y\!+\!\beta X Y^3\!+\!Z^2 \big (-\gamma X Y\!+\!(\omega ^2-1) X^2\!+\!1\big ) \big ]\\ \dot{Y}&= \dfrac{1}{Z^2} \Big [ (Y^2-1) Y(\alpha X^2 +\beta Y^2)\\&\quad \ + Z^2 \big (Y (-\gamma (Y^2-1)-X Y)+\omega ^2 X (Y^2-1)\big ) \Big ]\\ \dot{Z}&=\dfrac{Y}{Z} \big [\alpha X^2 Y+\beta Y^3+Z^2 \big ((\omega ^2-1) X-\gamma Y\big )\big ] \end{aligned} \end{aligned}$$

System (1) has eight equilibria on the equator $X^2+Y^2=1$ of $\mathrm {S}^2$ (see Theorem 1 from Chapter 3.10 of [46]) that represents the limit $x, y \rightarrow \infty$. In general, the equilibria are given by the solutions of the following equation:

3 $$\begin{aligned} XQ_m(X,Y)-YP_m(X,Y)=0 \end{aligned}$$

where $P_m$ and $Q_m$ are homogeneous *m*-th degree polynomials in *x* and *y* according to the following representation of the system (1):

4 $$\begin{aligned} \begin{aligned} \dot{x}&=P(x,y)=P_1(x,y)+\dots +P_m(x,y)\\ \dot{y}&=Q(x,y)=Q_1(x,y)+\dots +Q_m(x,y) \end{aligned} \end{aligned}$$

In our case, the highest degree homogeneous polynomials are:

5 $$\begin{aligned} \begin{aligned} P_3(x,y)&=0\\ Q_3(x,y)&=-\alpha x^2y-\beta y^3 \end{aligned} \end{aligned}$$

Hence, all equilibria at the equator are the solutions of the following system of equations:

6 $$\begin{aligned} \begin{aligned}&X^2+Y^2=1\\&XQ_3(X,Y)-YP_3(X,Y)=-\alpha X^3Y-\beta Y^4=0 \end{aligned} \end{aligned}$$

and are given by:

7 $$\begin{aligned} \begin{aligned} X&=0,&Y&=\pm 1,\\ X&=\pm 1,&Y&=0,\\ X&=\pm \dfrac{\sqrt{\alpha }}{\sqrt{\alpha -\beta }},&Y&=\pm \dfrac{\sqrt{\beta }}{\sqrt{\beta -\alpha }.} \end{aligned} \end{aligned}$$

The flow between the nodes is determined using the following equation (see Theorem 1 from Chapter 3.10 of [46]):

8 $$\begin{aligned} G_{m+1}=\cos \theta Q_m(\cos \theta ,\sin \theta )-\sin \theta P_m(\cos \theta ,\sin \theta )\!=\!0,\nonumber \\ \end{aligned}$$

where $\theta$ is an angle along the equator.

The flow between the equilibria on the equator of the Poincaré sphere is counterclockwise if $G_{m+1}>0$ and clockwise where $G_{m+1}<0$. We find that only the equilibria $X=0, Y=\pm 1$ are hyperbolic. They are stable nodes for $\beta <0$ and are unstable nodes for $\beta >0$. The other six equilibria as given in (7) are non-hyperbolic. We established their types by combining information gathered from the flow on the equator and from numerical integration of the transformed system (2). We summarise our findings in two representative cases in which, as the parameter $\gamma$ increases, the period and amplitude of the stable periodic orbit grows to infinity exponentially fast and the periodic orbit disappears. More specifically, at the critical value $\gamma ^*$, the stable periodic orbit becomes a heteroclinic cycle connecting four equilibria of saddle type at the equator on the Poincaré sphere.

In Fig. 2, we illustrate how the structure of the global phase portrait of the system (2), projected on the (*X*, *Y*)-plane, changes with increasing $\gamma$. Fig. 2a–c, for $\alpha =-1, \beta =1$, shows the transition occurring as $\gamma$ is varied in the bifurcation diagram of Fig. 1c; and Fig. 2d–f, for $\alpha =1, \beta =-0.1$ shows the transition occurring as $\gamma$ is varied in the bifurcation diagram of Fig. 1d. In both cases, the disappearance of the stable limit cycle solution in the model is due to the same mechanism. However, depending on the signs of the parameters $\alpha , \beta$, different invariant objects are involved in the transition. Panels (a–c) in Fig. 2 show that there are two types of connecting orbits in the phase space of the HKB oscillator. The first type connects the unstable equilibria $(0,\pm 1)$ (red dots) with the saddle points $(\pm 1,0)$ (black dots) and the second connects the saddle points $(\mp \sqrt{\alpha }/\sqrt{\alpha -\beta }, \pm \sqrt{\beta }/\sqrt{\beta -\alpha })$ (black dots) with the stable periodic orbit surrounding the unstable equilibrium at the origin (0, 0). As the parameter $\gamma$ increases, the two types of connections become tangent and the periodic orbit stretches along the *X* axis as depicted in panel (b) for value of $\gamma =3.65860608978$ (just before the transition). At the critical value, $\gamma =\gamma ^*$, the periodic orbit becomes a heteroclinic cycle connecting four saddle equilibria. After the transition, the heteroclinic cycle disappears and the global phase portrait changes. In panel (c), we show that after the transition there are connections between the saddle points $(\mp \sqrt{\alpha }/\sqrt{\alpha -\beta }, \pm \sqrt{\beta }/\sqrt{\beta -\alpha })$ and the stable equilibria $(\pm \sqrt{\alpha }/\sqrt{\alpha -\beta }, \pm \sqrt{\beta }/\sqrt{\beta -\alpha })$, and between the saddle points $(\pm 1,0)$ and the unstable equilibrium at the origin (0, 0). In this case, the single HKB oscillator has stable periodic solutions only for $\gamma \in (0, \gamma ^*)$. Panels (d, e) in Fig. 2 demonstrate that, for $\alpha =1, \beta =-0.1$, in addition to the stable periodic orbit there is also an unstable periodic orbit (red loop) surrounding the stable equilibrium at the origin (0, 0) (green dot). Although unstable periodic orbits could not be observed experimentally, such objects are important from dynamical systems point of view. For example, in this case the branch of unstable periodic orbits forms the boundary between the basins of attraction of the coexisting stable equilibrium and stable periodic orbit for $\gamma \in (\gamma _{\text {SN}}, \gamma ^*)$ (see Fig. 1d). Irrespective of the presence of unstable limit cycle, we find again two types of connecting orbits in the phase space for $\gamma <\gamma ^*$ as shown in panel (d). The first type connects the unstable equilibria $(\mp \sqrt{\alpha }/\sqrt{\alpha -\beta }, \pm \sqrt{\beta }/\sqrt{\beta -\alpha })$ (red dots) with the saddle points $(\pm \sqrt{\alpha }/\sqrt{\alpha -\beta }, \pm \sqrt{\beta }/\sqrt{\beta -\alpha })$ (black dots). The second type connects the saddle points $(\pm 1,0)$ (black dots) with the stable periodic orbit. Here we observe again that the connections become tangent to the periodic orbit, as it stretches along the *X*-axis growing into a heteroclinic cycle between four saddle equilibria (black dots) for $\gamma =\gamma ^*$, as depicted in panel (e) where $\gamma =-0.022618$ (just before the transition). After the transition $\gamma >\gamma ^*$, the heteroclinic cycle disappears and the invariant objects of the system reconnect. This, however, occurs in a different manner compared to the case presented in panels (a–c). The saddle equilibria $(\pm 1,0)$ are now connected with stable nodes $(0,\pm 1)$ and the unstable periodic orbit is connected to the saddle points $(\pm \sqrt{\alpha }/\sqrt{\alpha -\beta }, \pm \sqrt{\beta }/\sqrt{\beta -\alpha })$. In panel (f), we show the phase portrait for $\gamma =0.5$, which illustrates the connections after the unstable periodic orbit disappeared in a subcritical Hopf bifurcation (at $\gamma =0$, compare with Fig. 1). In this case, the single HKB oscillator has stable periodic solutions only for $\gamma \in (\gamma _\mathrm{SN},\gamma ^*)$.

### Bifurcation analysis of the full HKB model

#### Full system model equations

Previous analysis of the HKB model has focussed on the dynamics of the relative phase that is given by the difference of the two oscillators’ phases. However, in applications involving VP interaction environments [13, 60, 61], other properties of the HKB model dynamics become crucial. Such properties include the amplitude and phase of the oscillatory solutions, as well as their existence, parameter dependence and stability. In order to address these questions, we focus below on the full HKB system. The original HKB model evolves in time (measured in seconds) according to a set of nonlinear differential Equations [22]:

9 $$\begin{aligned} \ddot{x_1} + \dot{x_1} \left( \alpha x_1^2 + \beta \dot{x_1}^2 - \gamma \right) +\omega ^2 x_1= & {} I_{12}(\dot{x_1}, \dot{x_2}, x_1, x_2)\nonumber \\ \ddot{x_2} + \dot{x_2} \left( \alpha x_2^2 + \beta \dot{x_2}^2 - \gamma \right) +\omega ^2 x_2= & {} I_{21}(\dot{x_1}, \dot{x_2}, x_1, x_2),\nonumber \\ \end{aligned}$$

where $x_1$ and $x_2$ represent the position of the two agents’ end effectors and

10 $$\begin{aligned} I_{12}(\dot{x_1}, \dot{x_2}, x_1, x_2)= & {} (a+b(x_1-x_2)^2)(\dot{x_1}-\dot{x_2})\nonumber \\ I_{21}(\dot{x_1}, \dot{x_2}, x_1, x_2)= & {} (a+b(x_2-x_1)^2)(\dot{x_2}-\dot{x_1}), \end{aligned}$$

are coupling functions with coefficients $a, b \in \mathbb {R}$. The above system of two coupled second order ordinary differential equations (ODEs) (9) can be written as a four-dimensional autonomous system of first order ODEs:

11 $$\begin{aligned} \dot{x_1}&= y_1 \nonumber \\ \dot{x_2}&= y_2 \nonumber \\ \dot{y_1}&= (a+b(x_1-x_2)^2)(y_1-y_2) \nonumber \\&\quad - (y_1 \left( \alpha x_1^2 + \beta {y_1}^2 - \gamma \right) +\omega ^2 x_1)\nonumber \\ \dot{y_2}&= (a+b(x_2-x_1)^2)(y_2-y_1) \nonumber \\&\quad - (y_2 \left( \alpha x_2^2 + \beta {y_2}^2 - \gamma \right) +\omega ^2 x_2 ), \end{aligned}$$

where $x_i$ and $y_i$ represent position and velocity of the *i*th agent’s end effector, respectively. The resulting dynamical system has a four-dimensional state space [7]. The parameter $\omega$ (commonly referred to as *eigenfrequency*) defines, in conjunction with $\alpha , \beta$ and $\gamma$, the intrinsic dynamics of the two coupled oscillators. The oscillators’ positions and velocities are coupled via the parameters *a* and *b*, commonly referred to as *coupling strengths*. The HKB model behaviour then depends on the intrinsic dynamics parameters as well as the coupling strengths. Although coordination/synchronisation in system (11) emerges as a consequence of coupling, its dynamics (i.e. number, type and stability of coordination patterns) depends not only on the nature of the coupling but also on the intrinsic properties of each coupled oscillator. In the HKB system (11), both the intrinsic dynamics and the couplings are highly nonlinear, opening up the possibility of obtaining multi-stability and hence multi-functionality.

#### Coordination regimes in the HKB model

In this section, we study the existence and stability of the possible coordination regimes in the full HKB model (11) by conducting a systematic analysis in all model (control) parameters. The numerical bifurcation analysis is carried out using numerical continuation in AUTO [11]. We set $\texttt {NTST}=50$, $\texttt {NCOL}=4$ for the mesh, and $\texttt {EPSL}=10^{-9}$, $\texttt {EPSU}=10^{-9}$ for the tolerances of the Newton solver. We perform time-stepping simulations of the model (11) in MATLAB [39], using the ode45 solver with default numerical settings. In the simulations presented below, we use the following typical intrinsic dynamics parameter values as default, $\alpha =1, \beta =1, \gamma =1$ and $\omega =0.2$, unless otherwise stated in the figure legends.

**Fig. 3:**
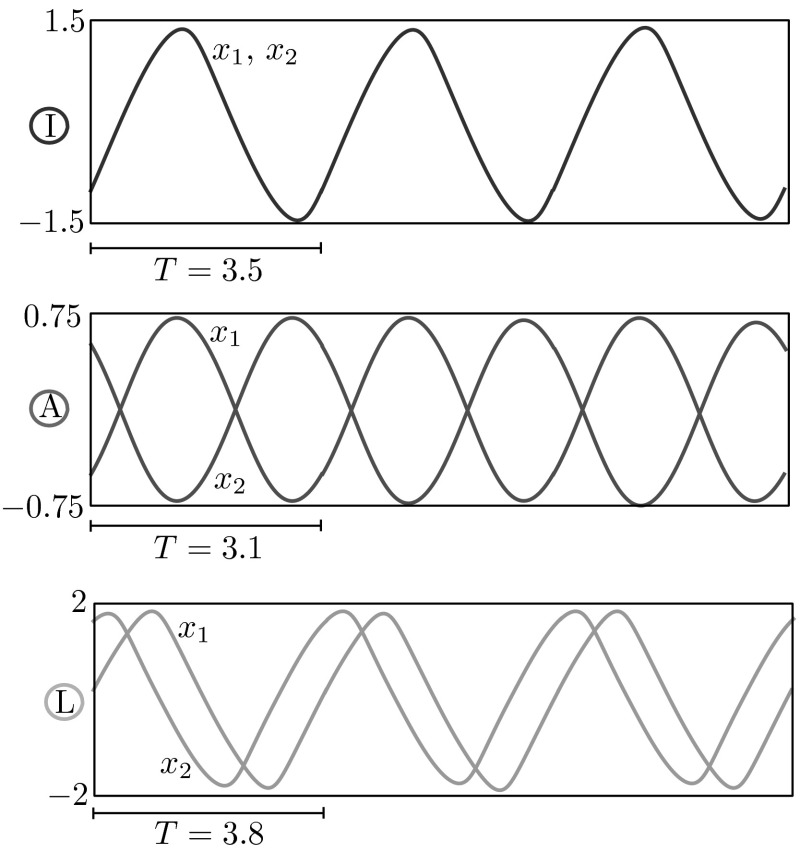
Examples of stable in-phase (I), anti-phase (A) and phase-locked (L) solutions. Solutions and parameter values are also indicated in the bifurcation diagrams of Fig. 4

**Fig. 4:**
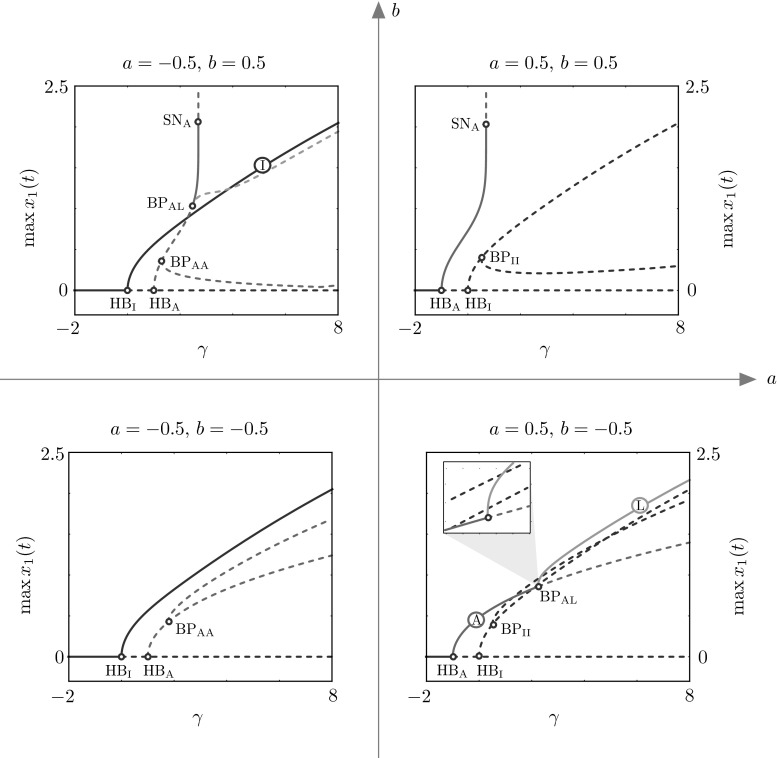
Representative bifurcation diagrams in the parameter $\gamma$ for all possible combinations of coupling strengths, *a* and *b*. *Solid* (*dashed*) lines represent stable (unstable) states of (11)

In agreement with previously performed analysis on the HKB relative phase dynamics [1, 3, 9, 17, 19, 22], we confirm existence and study the stability of the well-characterised in-phase and anti-phase oscillatory solutions. Moreover, we find a new family of stable periodic phase-locked solutions characterised by relative phase in the interval $(0^{\circ }, 180^{\circ })$. These solutions are found to be stable in a wide range of parameter values. We note that this family of solutions is unstable for the commonly used set of model parameters based on [22]. Examples of the three solution types described above are plotted in Fig. 3. We show how such solutions are born when we vary $\gamma$ for various combinations of the parameters *a* and *b* in the bifurcation diagrams of Fig. 4, whose branches are colour-coded as in Fig. 3. Here and henceforth, we use subscripts I, A, L (or combinations thereof) to indicate bifurcations occurring on solution branches of in-phase, anti-phase and phase-locked solutions, respectively. We also keep the corresponding colour-code convention for branches of solutions and solutions profiles of in-phase, anti-phase and phase-locked type.

**Fig. 5:**
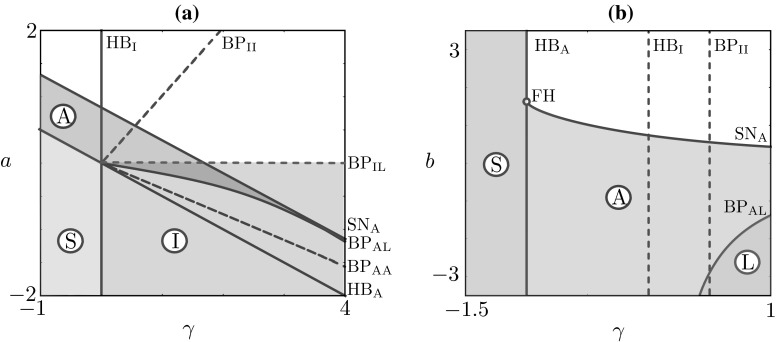
Two-parameter continuations of bifurcations occurring in Fig. 4. *Panel* **a** We fix $b=0.5$ and continue in the $(\gamma ,a)$-plane the bifurcations of the *top panels* of Fig. 4; *shaded areas* represent regions of stability for steady states (S), anti-phase (A) and in-phase (I) periodic solutions. *Panel* **b** We fix $a=0.5$ and continue in the $(\gamma ,b)$-plane the bifurcations in the right panels of Fig. 4; stable phase-locked solutions are indicated by (L)

In-phase and anti-phase coordination regimes are born via Hopf bifurcations (${\text {HB}}_\text {I}$, ${\text {HB}}_\text {A}$) of the trivial steady state. In the first quadrant (where the coupling strength parameters are both positive), anti-phase coordination is the only stable state: a branch of unstable in-phase solutions is born at ${\text {HB}}_\text {I}$ and bifurcates at a symmetry-breaking bifurcation, ${\text {BP}}_{\text {II}}$, giving rise to a secondary branch of unstable in-phase solutions where the oscillation amplitudes for agent 1 and 2 differ. In the third quadrant (where the coupling strength parameters are both negative), the scenario is specular: in-phase oscillations are now stable, while anti-phase solutions are unstable and bifurcate at ${\text {BP}}_{\text {AA}}$. In the first and third quadrants of the (*a*, *b*)-plane ($a=0.5$, $b=0.5$ and $a=-0.5$, $b=-0.5$, respectively), there are no branches of phase-locked solutions.

Phase-locked coordination regimes arise in the second and fourth quadrants of the (*a*, *b*)-plane, at symmetry-breaking bifurcations of anti-phase solutions (${\text {BP}}_{\text {AL}}$). Phase-locked solutions are found to be always stable (unstable) in the fourth (second) quadrant. We note that, in these quadrants, the coupling non-linearities $I_{12}$ and $I_{21}$, as functions of $x_1 - x_2$, attain both negative and positive values as opposed to what happens in the first and third quadrants, where such functions are strictly positive and strictly negative, respectively. Stable phase-locked solutions, spanning relative phases in the range of $(0^{\circ }, 180^{\circ })$, exist for $a>0$ and $b<0$. Such parameter settings could be used to model experiments displaying coordination regimes different from the canonical in-phase and anti-phase ones (see Discussion section for more details).

**Fig. 6:**
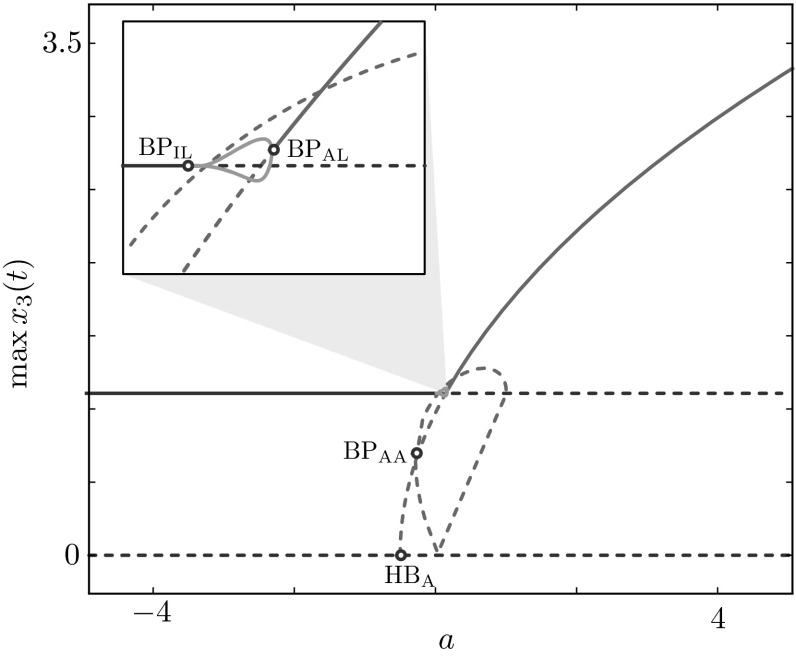
Continuation in the parameter *a*, for $b=-0.5$ and $\gamma =1$, $\omega =2$, $\alpha =\beta =1$. The branches show that, with suitable combination of the parameters, it is possible to have stable in-phase, anti-phase and phase-locked oscillations by varying *a*. *Solid lines* represent stable and *dashed lines* unstable states of (11)

In Fig. 5, we summarise the behaviour of the representative examples reported above, for selected values of *a* and *b*, by continuing in $(\gamma ,a)$ and $(\gamma ,b)$ all bifurcation points found in Fig. 4. The two-parameter continuations are performed so as to show how the solution landscape changes as we pass from the first to the second quadrant (continuation in $(\gamma ,a)$-plane) and from the first to the fourth quadrant (continuation in $(\gamma ,b)$-plane). In these two-parameter bifurcation diagrams, we highlight areas where stationary and oscillatory solutions are stable. In the $(\gamma ,a)$-plane, the organising centre is at $\gamma =0$, $a=0$: at this point, the eigenvalues of the linearised Jacobian at the trivial state $(x_1,x_2,x_3,x_4)=(0,0,0,0)$ are purely imaginary, equal to $\pm 2 i$, each with multiplicity 2, corresponding to eigenvalues $(0,\mp i/2,0,1)$ and $(\mp i/2,0,1,0)$. For low positive values of the damping $\gamma$, the system supports stable in-phase solutions (for negative values of *a*) and stable anti-phase solutions (in a wedge delimited by the locus of ${\text {SN}}_\text {A}$ and ${\text {BP}}_{\text {AA}}$). In a sizeable region of parameter space, stable in-phase and anti-phase solutions coexist (see intersection between magenta- and blue-shaded areas). We note that the original set of parameter values based on [22] could be found in this region.

**Fig. 7:**
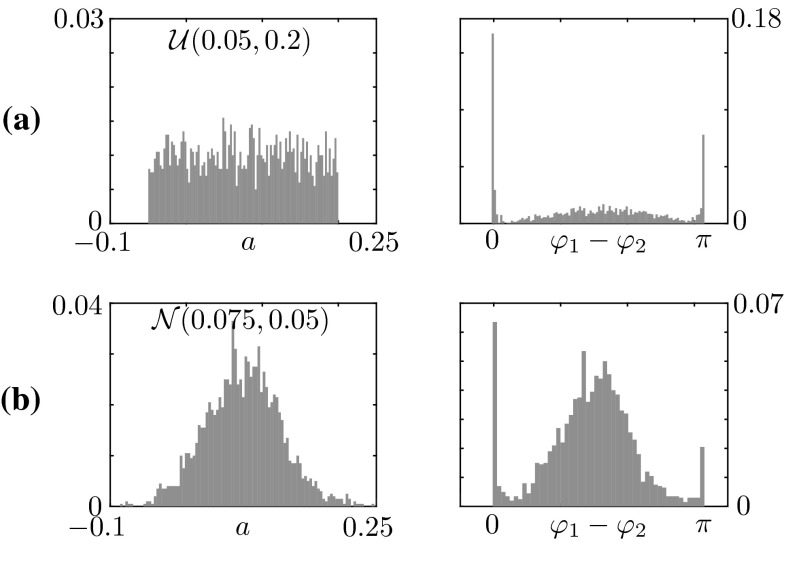
Distribution of the phase difference between $x_1(t)$ and $x_2(t)$ (*left panels*) obtained when the control parameter *a* is randomly distributed (*right panels*) with values close to the inset of Fig. 6. **a** The parameter *a* is sampled from a uniform distribution (*left*) and 2000 independent simulations are performed; the histogram for the relative phase $\varphi _1 - \varphi _2$ is bimodal and sharply peaked around $0^\circ$ and $180^\circ$, with a small but nonzero probability of finding an intermediate phase lag. **b** The experiment is repeated with a normal distribution, which causes a third peak to develop around $90^\circ$ in the distribution for the phase lag; the latter peak is inherited from the distribution of the control parameter *a*

In the $(\gamma ,b)$-plane, the organising centre is a fold–Hopf bifurcation around $\gamma =-1$, $b\approx 1.625$ (FH in Fig. 5) where the locus of saddle nodes of the anti-phase solutions, ${\text {SN}}_\text {A}$, collides with the locus of Hopf bifurcations ${\text {HB}}_\text {A}$. In this region of parameter space, phase-locked solutions are found for sufficiently high damping and sufficiently negative values of *b*. It should be noted, however, that phase-locked and anti-phase oscillations do not coexist for the default choice of intrinsic dynamics parameter values. As it can also be verified analytically, the locus of bifurcations ${\text {HB}}_\text {A}$ and ${\text {HB}}_\text {B}$ of the stationary steady state do not depend on *b*. It should be also noted that, for suitable combination of the parameters, it is possible to visit stable in-phase, anti-phase and phase-locked oscillations by varying *a*. An example of continuation in *a* for $b=-0.5$ and $\gamma =1$, $\omega =2$, $a=b=1$ is depicted in Fig. 6. It can be clearly seen in the inset of Fig. 6 that, as *a* increases, the stable in-phase coordination regime (characterised by relative phase $0^{\circ }$) loses stability; then, a phase-locked coordination regime emerges (ranging over relative phases in $(0^{\circ }, 180^{\circ })$ in a continuous fashion) and eventually a stable anti-phase coordination regime (characterised by relative phase $180^{\circ }$) is established. The bifurcation diagram implies that, in an experimental set-up where *a* were to be assigned randomly, we would observe trajectories with relative phases distributed in the interval $(0^{\circ }, 180^{\circ })$, and with peaks at $0^\circ$ and $180^\circ$. To verify this prediction, we performed an uncertainty quantification study, in which *a* is assigned randomly, near the inset of Fig. 6, and histograms of relative phases between $x_1(t)$ and $x_2(t)$ are computed a posteriori. In Fig. 7a, we perform 2000 independent simulations, where *a* is sampled from the uniform distribution between 0.05 and 0.2 and plot the resulting phase lag histogram: the distribution for the phase difference $\varphi _1 - \varphi _2$ (right) is bimodal and sharply peaked around $0^\circ$ and $180^\circ$, as expected, with a small but nonzero probability of finding an intermediate phase lag. The likelihood that experiments display such intermediate relative phases is deeply affected by the distribution of *a*: if we pass from a uniform to a normal distribution for *a*, (Fig. 7b), the resulting phase lag distribution develops also a peak around $90^\circ$, which is inherited from the parameter distribution.

**Fig. 8:**
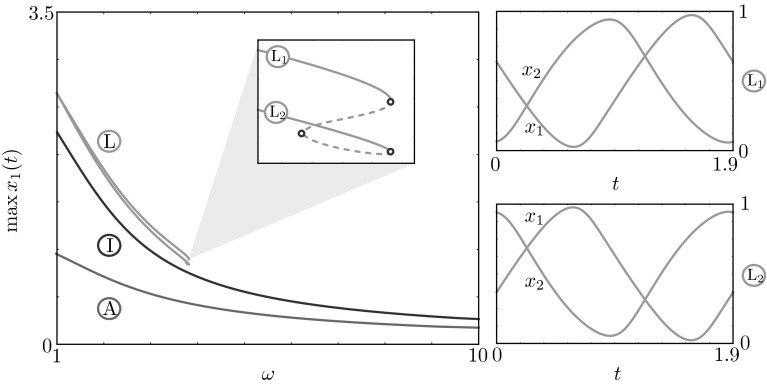
Continuation of in-phase, anti-phase and phase-locked solutions in the frequency $\omega$. Solutions behave similarly to the single HKB oscillator case (Fig. 1b), except they have various phase behaviours. The branch of phase-locked solutions undergoes a series of saddle-node bifurcation, giving rise to stable solutions in which the phase difference is reversed (see solutions profiles $\mathrm {L}_{1,2}$). We note that the branches in this figure do not coexist, as they are found in different regions of parameter space: $\alpha =\beta =1$ and $a=-0.5$, $b=-0.5$, $\gamma =5$ (in-phase), $a= 0.5$, $b=-0.5$, $\gamma =1.2$ (anti-phase) and $a= 0.5$, $b=-0.5$, $\gamma =6.2$ (phase-locked)

It is interesting to study the behaviour of various periodic solutions as the common eigenfrequency of the oscillators, $\omega$, varies. We selected stable in-phase, anti-phase and phase-locked solutions and continued them in $\omega$ (Fig. 8). We found that such solutions behave essentially as in the single oscillator case (Fig. 1b): low frequencies elicit large amplitude oscillations with abrupt time transitions, whereas large frequencies induce smoother small amplitude oscillations. In this case the changes in the oscillation patterns occur to both agents, with various phase differences. The branch of phase-locked solutions undergoes a series of saddle-node bifurcations, giving rise to stable solutions in which the phase difference is reversed (see solutions profiles $\mathrm {L}_{1,2}$ in Fig. 8).

**Fig. 9:**
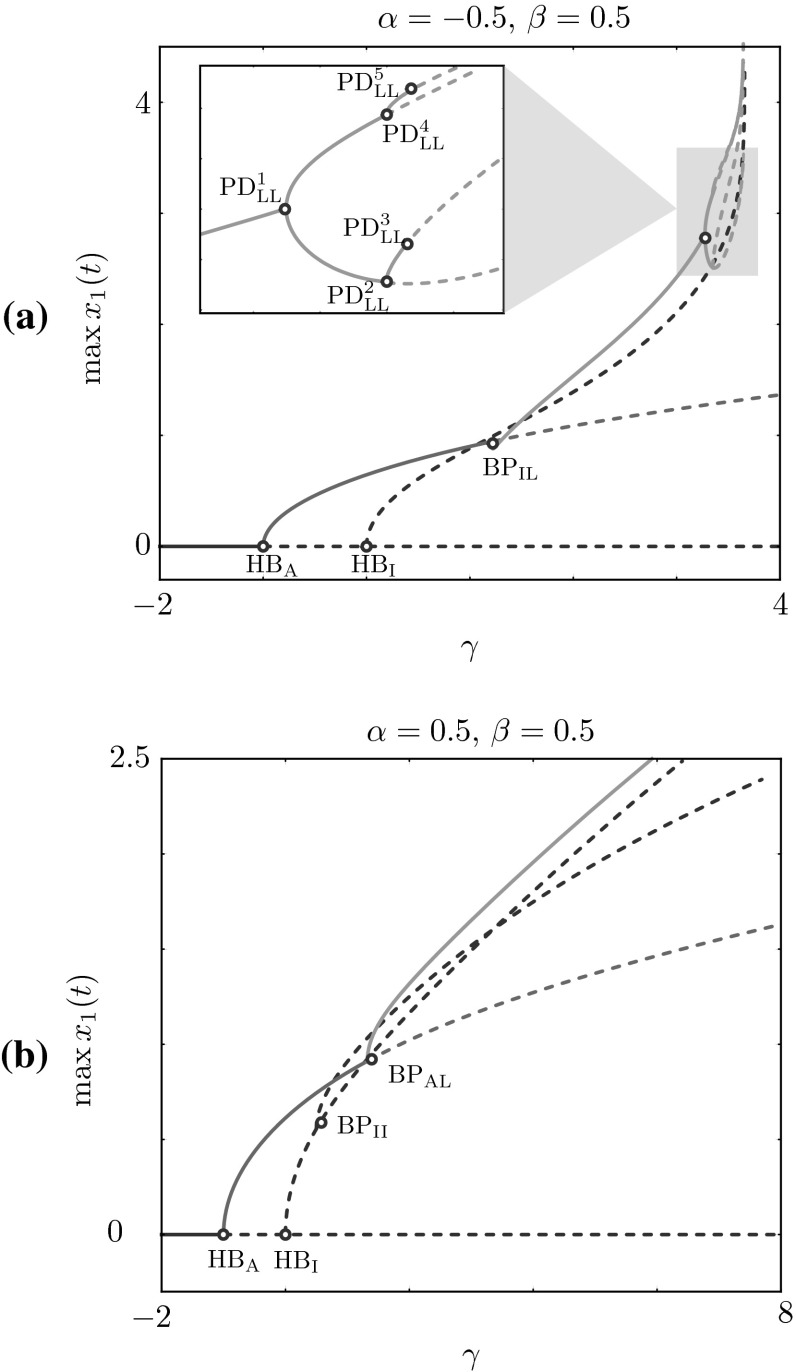
Bifurcation diagram in $\gamma$ for $\omega =2$, $a = 0.5$, $b=-0.5$ and various values of $\alpha$ and $\beta$. *Solid lines* represent stable and *dashed lines* unstable states of (11)

**Fig. 10:**
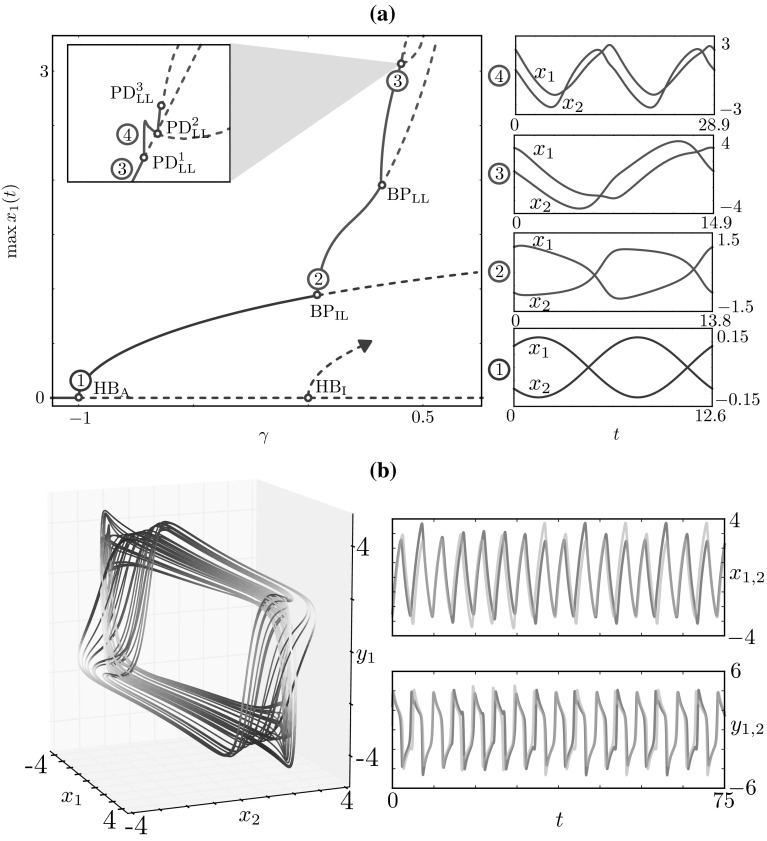
Period-doubling cascade. **a** Branch with period-doubling cascade and stable non-trivial periodic solutions. We plot one period of several stable solutions along the branch, whereas we omit the unstable branch emanating from ${\text {HB}}_\text {I}$. Solutions feature increasing solution periods, $T_1 \approx 12.6$, $T_2 \approx 13.8$, $T_3 \approx 14.9$, $T_4 \approx 28.9$, corresponding to $\varOmega _1 \approx 0.500$, $\varOmega _2 \approx 0.455$, $\varOmega _3 \approx 0.422$, $\varOmega _4 \approx 0.217$, respectively. Parameters $\omega =0.5$, $a = 0.5$, $b=-0.5$ $\alpha =0.5$ and $\beta =-0.05$. **b** Attractor found for $\gamma =3.42$; the coordination regime shows erratic phase changes, during which $x_1$ and $x_2$ alternate in the leading position. This regime involves fast velocity switches, as evidenced by the time traces of $y_1$ and $y_2$. *Solid lines* represent stable and *dashed lines* unstable states of (11)

Finally we investigate the impact of intrinsic oscillator dynamics on the collective behaviour of the HKB model by performing bifurcation analysis in the intrinsic dynamics parameters $\alpha$ and $\beta$. Instead of presenting two-parameter bifurcation diagrams for different cases, we report here only notable examples of our computations (see Figs. 9 and 10a). The bifurcation structures found in these cases have common traits with the ones discussed above for the coupling strengths parameters *a* and *b*, that is, the trivial steady state undergoes Hopf bifurcations to anti-phase and in-phase periodic states, and various symmetry-breaking bifurcations give rise to phase-locked solutions. Interestingly, when varying $\alpha$ and $\beta$ we could find period-doubling cascades, which are found robustly when $\alpha$ and $\beta$ have opposite signs, as evidenced in Fig. 9a, where $\alpha =-0.5$, $\beta = 0.5$, and Fig. 10a, where $\alpha =0.5$, $\beta = -0.05$. Representative stable solutions on the period-doubling cascade are also shown in Fig. 10a.

Using direct numerical simulations we explored the system behaviour close to the period-doubling cascade, finding chaotic regimes (see Fig. 10b) in which the solution remains bounded and features sudden erratic phase transitions, during which the agents alternate as leaders and followers. In this regime, the velocities $y_1$ and $y_2$ undergo fast switches. The existence of such complex solutions is perhaps not surprising from a dynamical systems viewpoint; however, the behaviour described above has not been reported nor investigated previously, and can be used to model experiments where the movement coordination is irregular in nature. Last but not least, knowledge about the existence of such solutions is critical when designing virtual player interaction environments [34, 59–61] and/or planning human dynamic clamp experiments based on the HKB model [13].

#### Bistability and hysteresis

**Fig. 11:**
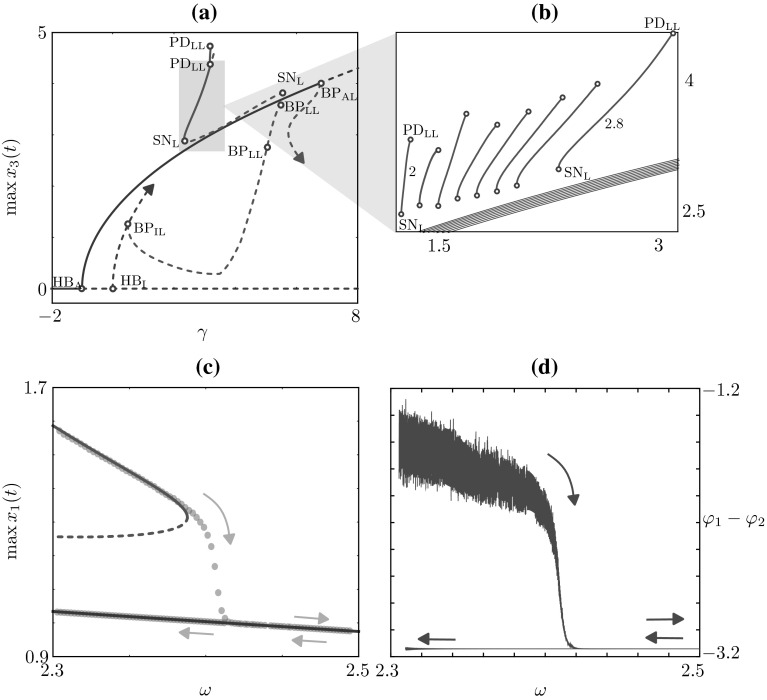
Bistability and hysteresis between anti-phase and phase-locked solutions. **a** The in-phase branch (*blue*) undergoes a symmetry-breaking bifurcation (${\text {BP}}_{\text {IL}}$) and the resulting unstable phase-locked branch, featuring two further symmetry-breaking bifurcations (${\text {BP}}_{\text {IL}}$), restabilises at a saddle-node bifurcation, before a period-doubling cascade takes place. A stable portion of the phase-locked branch (*solid green line* between ${\text {SN}}_\text {L}$ and ${\text {PD}}_{\text {LL}}$) coexists with the anti-phase branch originating at ${\text {HB}}_\text {I}$ (*solid red branch*). Parameters: $a=0.5$, $b=0.5$, $\omega =3$, $\alpha =-1.7$, $\beta =0.5$. **b** We repeat the experiment for $\omega \in [2,2.8]$ and plot stable branches to highlight the bistability region. **c** $\omega$ is varied by continuation and by quasi-static sweeps in direct numerical simulations (*blue dots*), for $\gamma =1.7$; the time simulation follows the phase-locked branch up to the saddle node at $\omega \approx 2.4$, where an abrupt and hysteretic transition to an anti-phase solution is observed. **d** Phase lag during numerical simulation in **c** (colour figure online)

In this section, we explore further the dependence of the HKB model dynamics on the intrinsic properties of the coupled oscillators. In suitable regions of parameter space we find coexisting stable periodic states characterised by different relative phases or phase lags. In Fig. 11a, we run a continuation similar to the ones presented above, but we set $\alpha =-1.7$. The branches of this bifurcation diagram are qualitatively similar to the ones of the previous sections; however, the in-phase periodic branch originating at the Hopf bifurcation ${\text {HB}}_\text {I}$ undergoes a symmetry-breaking bifurcation (${\text {BP}}_{\text {IL}})$. Such branch is initially unstable, undergoes 2 other symmetry-breaking bifurcations, restabilises at a saddle-node bifurcation and then features a period-doubling cascade. The stable portion of this branch (solid green branch between ${\text {SN}}_\text {L}$ and ${\text {PD}}_{\text {LL}}$) coexists with a branch of stable anti-phase solutions originating from the trivial state at ${\text {HB}}_\text {A}$ (red branch).

This bifurcation structure opens up the possibility of observing abrupt relative phase transitions between phase-locked (at any relative phase between $0^{\circ }$ and $180^{\circ }$) and anti-phase (at relative phase equal to $180^{\circ }$) coordination regimes as a function of the eigenfrequency $\omega$. We find that bistability is observed in a significant region of parameter space: in the inset of Fig. 11b we report overlapping stable portions of phase-locked and anti-phase branches as we vary the eigenfrequencies $\omega$. As $\omega$ is varied, the anti-phase branch (red) changes only slightly, while the stable phase-locked branch moves to the left and expands. Our analysis predicts coexistence in the region $(\gamma ,\omega ) \in [1.2,3.2]\,\times \,[2,2.8]$ (which was found robustly in other parameter regions, not shown). It is important to note that this phase transition is qualitatively different from the transition addressed by the original HKB model [22], where an increase in frequency leads to transition from anti-phase to in-phase coordination. In the parameter regime described above, an increase in frequency leads to transition from phase-locked to anti-phase coordination behaviour.

To illustrate the dynamical switch between solution types, we perform time-stepping simulations in which the eigenfrequency $\omega$ is varied quasi-statically and compare with the bifurcation analysis. In Fig. 11c, we continued an anti-phase (red) and phase-locked (green) solution for $\gamma =1.7$, $\omega =2.3$ in the parameter $\omega$; the phase-locked branch destabilises at a saddle-node bifurcation, whereas the anti-phase branch remains stable for $\omega \in [2.3,2.5]$. We then initialised a time simulation on the phase-locked branch (blue dots in Fig. 11c) and changed $\omega$ in slow increments (from $\omega = 2.3$ up to $\omega =2.5$) followed by small decrements (from $\omega = 2.5$ down to $\omega =2.3$). The time simulation shows an abrupt and hysteretic change in the solution type. This could be further appreciated in Fig. 11d where we plot the time simulation using the phase lag $\varphi _1 - \varphi _2$. The $x_2$ is delayed with respect to $x_1$, with an initial phase lag $\varphi _1-\varphi _2 \approx 90^{\circ }$; when $\omega \approx 2.4$, we observe a transition to an orbit with $\varphi _1 - \varphi _2 \approx 180^{\circ }$ (anti-phase solution).

#### Effect of heterogeneity in eigenfrequencies on the coordination regimes

**Fig. 12:**
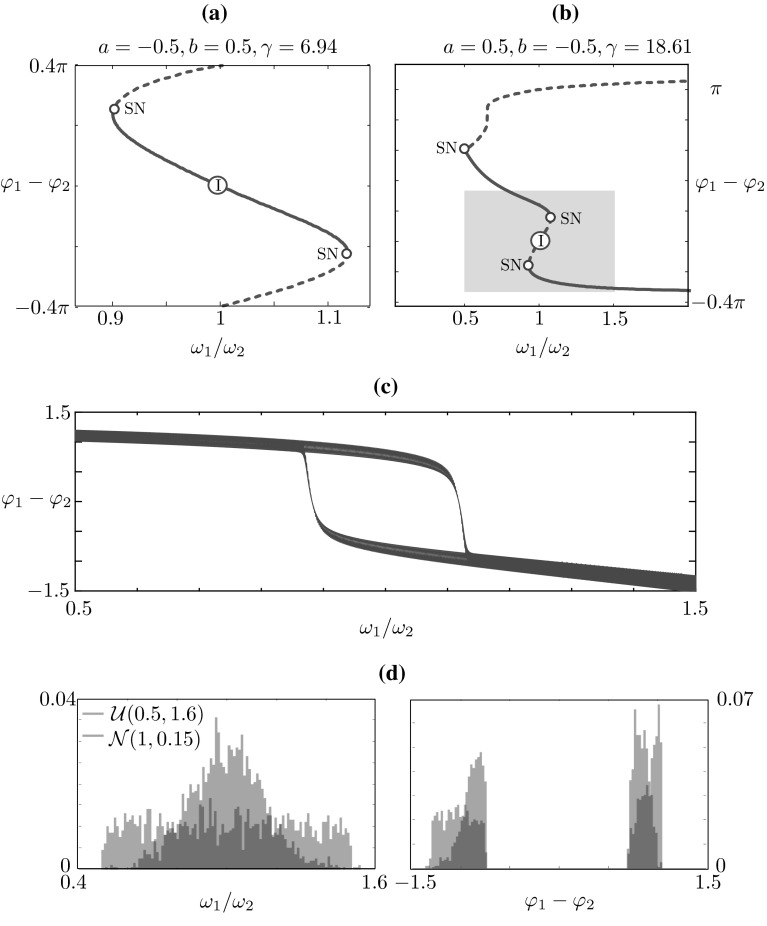
Phase difference between periodic solutions $x_1(t)$ and $x_2(t)$ as a function of the ratio $\omega _1/\omega _2$. **a** A stable solution on the in-phase branch in the fourth quadrant of Fig. 4 is continued in $\omega _1/\omega _2$. **b** The continuation is repeated starting from a solution on the phase-locked branch in the second quadrant of Fig. 4. In both cases, heterogeneity in the eigenfrequencies impacts the phase lag of the solution. **c** When the ratio $\omega _1/\omega _2$ is modulated with a slowly varying sinusoidal function, the actors alternate in the leading position with hysteretic cycles, which follow the branches in the *inset* of **b** and jump at the corresponding saddle-node bifurcations. **d** We perform an experiment similar to the one in Fig. 7; when the parameter $\omega _1/\omega _2$ is drawn randomly near the *shaded area* in **b** from a uniform (*blue*) or a normal (*red*) distribution, the resulting phase lag distribution is bimodal, with peaks at $\pm 57.30^\circ$, as predicted by the bifurcation diagram in **b** and by the parameter sweep in **c** (colour figure online)

In the computations shown so far, the two oscillators possess a common eigenfrequency $\omega$. In order to study the effect of heterogeneities on coordination, we introduce two parameters $\omega _1$, $\omega _2$, then fix $\omega _1$ to the nominal value $\omega _1=2$ and use the ratio $\omega _1/\omega _2$ as a continuation parameter. The difference in eigenfrequencies introduces a heterogeneity in the system and has the potential to turn in-phase solutions into phase-locked solutions and vice-versa. In order to illustrate this idea, we performed bifurcation analysis in the parameter $\omega _1/\omega _2$ investigating the in-phase solutions which exist for parameter values within the range of those used in the original HKB model [22], as well the stable phase-locked solutions which we reported above for $a>0$ and $b<0$ (see Fig. 4). In Fig. 12, we initialise the continuation with an in-phase and a phase-locked periodic solution. We plot the bifurcation diagram in terms of the phase lag (measured in radians), by computing the approximate phases times $\varphi _i = t_i/T$, for $i=1,2$, where $t_i$ is the time at which the orbit $x_i(t)$ attains its maximum and *T* is the solution period. Fig. 12a depicts a stable, initially in-phase, solution at $\omega _1/\omega _2=1$ that turns into a phase-locked solution as $\omega _1/\omega _2$ is increased/decreased, losing stability at saddle-node bifurcation. In Fig. 12b, we show how the phase lag is reduced when the frequency ratio is varied and an in-phase (albeit unstable) solution is eventually attained, before a new phase-locked solution arises.

The bifurcation structure in Fig. 12b implies that hysteresis between phase-locked solutions with opposite phase lags (relative phases) is possible in the model. To illustrate this, we perform time-stepping simulations in which the ratio is varied quasi-statically as $\omega _1(t) = \omega _2(t)[1 + \sin (0.005 t)]$ and plot the results in Fig. 12c. The two oscillators swap in the leader and follower role, following the branches of Fig. 12b and switching roles at the corresponding saddle-node bifurcations. This numerical experiment could be interpreted in the light of the joint improvisation scenario in the “mirror game”, a recently proposed paradigm for studying the dynamics of two people improvising motion together [45]. In particular, as the participants are asked to imitate each other and create synchronised and interesting motions, they would be naturally trying to adjust their movement velocities and thus eigenfrequencies to each other. This would lead to variation in the ratio of their eigenfrequencies and, respectively, exchange of leader and follower roles while playing the game. Indeed, observations based on our data collected in a “mirror game” setting [55] indicate that the distribution of relative phase during a typical joint improvisation sessions are bimodal pointing to possible hysteretic dynamics. As we see in Fig. 12d, the bimodal distribution emerges also in the case of randomly assigned frequency ratio $\omega _1/\omega _2$: when the value of this parameter is drawn randomly (close to the hysteretic region) from a uniform or a normal distribution, the resulting phase lag distribution is bimodal, with peaks at $\pm 57.30^\circ$, as predicted by the bifurcation diagram in Fig. 12b and by the parameter sweep in Fig. 12c.

## Discussion

In this paper, we have systematically investigated the dynamics of the HKB model in the state space spanned by the position and velocities of the coupled oscillators. Furthermore, we go beyond the weakly coupled regime and consider the coupling strength parameters as generic. We show that stable periodic solutions in the single HKB oscillator model are born via a Hopf bifurcation as the damping parameter $\gamma$ becomes positive. Furthermore, we reveal that under certain intrinsic oscillator properties the periodic solutions of the single HKB model could disappear via a heteroclinic cycle, associated with rapid increase in the magnitude of the state variables. Although such behaviour cannot be observed in a physical system, it can have significant consequences for the design and development of the virtual players. Bifurcation analysis of the full four-dimensional HKB model reveals a variety of different coordination dynamics. Attractors at a constant relative phase of $0^{\circ }$ (in-phase) and/or $180^{\circ }$ (anti-phase) are born via Hopf bifurcations detected in the damping parameter $\gamma$. We find symmetrical attractors of phase-locked solutions at intermediate values of relative phase (between $0^{\circ }$ and $180^{\circ }$), which increase or decrease gradually as $\gamma$ is increased. We demonstrate that the phase-locked solutions are born in a symmetry-breaking bifurcation of periodic orbit in which the anti-phase periodic attractor loses stability as the damping parameter $\gamma$ is varied. Changing the sign of the coupling strengths has the effect of shifting the attractors by $180^{\circ }$, thus changing the phase that remains stable at high frequencies, from $0^{\circ }$ to $180^{\circ }$ or vice-versa. We also show that change in the intrinsic oscillators’ properties (i.e. varying the parameter $\alpha$) can lead to complex dynamics mediated via a period-doubling cascade. Furthermore, different intrinsic dynamics can also bring about a variety of bistability modes, which are different that the type of bistability described in the original HKB model study [22]. Finally we consider a case of a heterogeneity in the system by introducing difference in the eigenfrequency of the coupled oscillators. We demonstrate how this results in bistability and hysteresis. Our uncertainty quantification simulations presented in Fig. 12d confirm that in the case of heterogeneous oscillators hysteresis loops and phase-locked coordination modes should be expected in experiments, as suggested in [2]. What is more, existence of such hysteresis loop provides an excellent opportunity for a quantitative experimental validation of the HKB model using two heterogeneous coupled oscillators, e.g. by putting weights on body parts as suggested in [2] or by using heterogeneous pendula as in [50].

In a large number of multi-stable examples observed experimentally, the patterns of stability change under different conditions. Bimanual finger coordination is bistable at low frequencies, but above a critical frequency the anti-phase pattern is no longer sustainable [22]. Similarly postural sway is bistable at low frequencies ($20^{\circ }$ and $180^{\circ }$), but the phase-locked ($20^{\circ }$) mode looses stability at high frequencies or when other behaviours, such as reaching, are incorporated in the task [2]. These transitions between stable states, and particularly the loss of stability of the anti-phase mode at high frequencies, appear to be a fundamental feature of human coordination [33]. The hypothesis that these real-world patterns and transitions between them are emergent phenomena due to a self-organised dynamical system are substantiated by experimental results such as critical fluctuations, critical slowing down and hysteresis between modes [2, 18, 51]. In this paper, we make the first step towards identifying parameter regimes and dynamics that would allow to model a variety of different experimental observations using the same modelling framework.

Many recent experimental studies of human movement coordination [5, 10, 12, 15, 24, 57] have reported persistent movement coordination dynamics other than the well-known in-phase and anti-phase synchronisation behaviour that have inspired the development of the HKB model [22]. Despite the large number of behaviours whose dynamics are well represented by the theoretically predicted in-phase and anti-phase stability, there are several counter examples where human body movements show evidence of stability at different or additional intermediate phases. Examples of real-world systems with stabilities at other relative phases include: the human postural system (stability at $20^{\circ }$) [2], amble to walk gait in quadrupeds (stability at $90^{\circ }$) [8], the bipedal skipping gait [42], coordination tendencies of successful defences ($30^{\circ }$) and unsuccessful defences ($90^{\circ }$) in soccer [12], squash ($135^{\circ }$) [41] and butterfly stroke swimming ($90^{\circ }$) [14] as well as variety of relative phase distributions in other team sports [10]. There is evidence that other phases can be stable simultaneously with $0^{\circ }$ and $180^{\circ }$. These multi-stable dynamics can exist naturally or be learnt [57]. Our results about the existence of stable phase-locked periodic solutions in the HKB model that span all possible relative phases between $0^{\circ }$ and $180^{\circ }$ could be related to some of the above mentioned experimental observations. In particular, analysis of the data collected from interactions between player dyads allowed for a description of the space-time dynamics of basketball match play. In the longitudinal direction, a strong attraction to in-phase was reported for all possible dyads but not so for the lateral direction. Instead, attractions to in-phase or anti-phase were observed among most dyads with the player vs. player dyads tending on balance to demonstrate less pronounced attractions or repulsions to certain relative phases than the player–opponent dyads [5, 15]. Interpersonal coordination tendencies of 1-vs-1 subphases were investigated in [12]. The experimental results presented in Fig. 2 in [12] could be, for example, qualitatively accounted for by the type of coordination stability dependence on the coupling parameter *a* found in the HKB model. Specifically (see Fig. 6 for $b=-0.5$ and $\gamma =1$, $\omega =2$, $\alpha =\beta =1$), as the coupling strength between the velocity components of the two oscillators increases, the stable coordination regime exhibited by the HKB model undergoes transitions from stable in-phase coordination for $a<0$ through stable phase-locked coordination spanning all possible relative phases in $(0^{\circ },180^{\circ }$); as *a* increases, to a stable anti-phase coordination regime with increasing amplitudes.

Last but not least, very recent experiments involving the use of virtual partner interaction [13, 34, 59–61] have employed to various degree the HKB model in order to study social interactions and interpersonal coordination. These studies have used adaptation in the HKB parameter values in their implementations. Knowledge about how the type and stability of the possible HKB model solutions depend on the model parameters could greatly facilitate the design and ensure robustness of such hybrid systems where a human interacts with a virtual partner whose movements are driven by the HKB model. Furthermore, comparison of the theoretical predictions and dynamics observed in experiments with a virtual partner could allow for quantitative, rather than qualitative, validation of different models of motor coordination. Although deficits of the HKB model are well known, see, for example, discussion in [3], our analysis demonstrates that this model has much richer dynamics than previously considered and showcases mathematical tools that could be very useful in future studies of human movement coordination.
